# The Achieving Self-directed Integrated Cancer Aftercare Intervention for Detection of Recurrent and Second Primary Melanoma in Survivors of Melanoma: Pilot Randomized Controlled Trial

**DOI:** 10.2196/37539

**Published:** 2022-09-08

**Authors:** Peter Murchie, Lynda Constable, Susan Hall, William Brant, Julia Allan, Marie Johnston, Judith Masthoff, Amanda Lee, Shaun Treweek, Dolapo Ayansina, Charlotte Proby, Kaz Rahman, Fiona Walter, Nigel Burrows, Amer Durrani, Graeme Maclennan

**Affiliations:** 1 Institute of Applied Health Sciences University of Aberdeen Aberdeen United Kingdom; 2 NHS Grampian Aberdeen United Kingdom; 3 Department of Information and Computing Sciences Universiteit Utrecht Utrecht Netherlands; 4 Molecular and Clinical Medicine School of Medicine University of Dundee Dundee United Kingdom; 5 Wolfson Institute of Preventive Medicine and Institute of Population Health Sciences London United Kingdom; 6 The Primary Care Unit, Department of Public Health and Primary Care University of Cambridge Cambridge United Kingdom; 7 Cambridge University Hospitals NHS Foundation Trust Cambridge United Kingdom

**Keywords:** primary care, melanoma, cancer, randomized controlled trial, survivorship, self-directed care, eHealth, Achieving Self-directed Integrated Cancer Aftercare, ASICA, well-being, quality of life, mobile phone

## Abstract

**Background:**

Melanoma is common with increasing incidence. Guidelines recommend monthly total skin self-examinations (TSSEs) by survivors to detect recurrent and new primary melanomas. TSSE is underperformed despite evidence of benefit.

**Objective:**

This study compares the effect on psychological well-being and TSSE practice of a self-directed digital intervention with treatment as usual in patients treated for a first stage 0 to IIC primary cutaneous melanoma within the preceding 60 months.

**Methods:**

This randomized clinical trial was conducted at 2 UK National Health Service hospitals (Aberdeen Royal Infirmary, Grampian, and Addenbrooke’s, Cambridge). Adults (≥18 years) diagnosed with a first 0 to IIC primary cutaneous melanoma were randomized to receive Achieving Self-directed Integrated Cancer Aftercare (ASICA), a tablet-based intervention prompting and supporting TSSE in survivors of melanoma, or to usual care. The hypothesis was that ASICA would increase TSSE practice in users affected by melanoma and compared with controls without affecting psychological well-being. The main primary outcomes were melanoma worry (Melanoma Worry Scale), anxiety and depression (Hospital Anxiety and Depression Scale), and quality of life (EQ-5D-5L) as well as secondary outcomes collected using postal questionnaires 3, 6, and 12 months following randomization.

**Results:**

A total of 240 recruits were randomized (1:1) into the ASICA (n=121, 50.4%) or control (n=119, 49.6%) groups. There were no significant differences between groups for melanoma worry at 12 months (mean difference: 0.12, 95% CI −0.6 to 0.84; *P*=.74), 3 months (0.23, 95% CI −0.31 to 0.78; *P*=.40), or 6 months (−0.1, 95% CI −0.7 to 0.51; *P*=.76). The ASICA group had lower anxiety scores at 12 months (−0.54, 95% CI −1.31 to 0.230; *P*=.17), 3 months (−0.13, 95% CI −0.79 to 0.54; *P*=.71), and significantly at 6 months (−1.00, 95% CI −1.74 to −0.26; *P*=.009). Depression scores were similar, being lower at 12 months (−0.44, 95% CI −1.11 to 0.23; *P*=.20) and 3 months (−0.24, 95% CI −0.84 to 0.35; *P*=.42) but only significantly lower at 6 months (−0.77, 95% CI −1.41 to −0.12; *P*=.02). The ASICA group had significantly higher quality of life scores at 12 months (0.044, 95% CI 0.003-0.085; *P*=.04) and 6 months (0.070, 95% CI 0.032-0.107; *P*<.001) and nonsignificantly at 3 months (0.024, 95% CI −0.006 to 0.054; *P*=.11). ASICA users reported significantly more regular (>5) TSSEs during the study year and significantly higher levels of self-efficacy in conducting TSSE. They also reported significantly higher levels of planning and intention to perform TSSE in the future.

**Conclusions:**

Using ASICA for 12 months does not increase melanoma worry, can reduce anxiety and depression, and may improve quality of life. ASICA has the potential to improve the well-being and vigilance of survivors of melanoma and enable the benefits of regular TSSE.

**Trial Registration:**

ClinicalTrials.gov NCT03328247; https://clinicaltrials.gov/ct2/show/NCT03328247

**International Registered Report Identifier (IRRID):**

RR2-10.1186/s13063-019-3453-x

## Introduction

### Background

The COVID-19 pandemic has seen the rapid deployment of digital technologies to manage both acute and scheduled health care with apparent success [1]. In the United Kingdom and elsewhere, digital technology has been deployed widely to manage triage and direct care to appropriate places and times [1]. Although demonstrating the great potential of digital health care across the National Health Service (NHS), uncertainties about the true impact on patients’ well-being and outcomes remain, and the rigorous development and evaluation of digital technologies has never been more urgent [2]. A particular area where digital technology could have much to offer is secondary prevention of cutaneous melanoma [3].

Melanoma is common, with approximately 16,200 people in the United Kingdom diagnosed each year, and its incidence has increased 5-fold in 30 years [4]. The UK guidelines recommend that patients treated for cutaneous melanoma receive extended hospital follow-up to detect recurrence or new primaries [5]. However, delivering melanoma follow-up to the growing population of survivors is burdensome for both individuals and health services [6]. Nevertheless, follow-up is important as approximately 20% of patients with early-stage melanoma experience a recurrence, and 4% to 8% develop a new primary, the risk of both being highest in the first 5 years [7-10]. Melanoma recurrence can present locally, regionally, or with distant metastases, and new primaries can occur anywhere [11]. Successful treatment of recurrent melanoma with targeted and immunological treatments is leading to significant improvements in survival even in advanced melanoma [12].

Therefore, it is important to detect new primary and recurrent melanomas in a timely way. Most recurrences and new primaries are detected by patients between scheduled follow-up visits [5]. Thus, guidelines recommend that patients conduct monthly total skin self-examinations (TSSEs; thorough checks of the total surface of the skin) during follow-up. A randomized trial in the United States showed that increasing TSSE practice for 6 months in the short term resulted in significantly more detection of potential melanoma in people with increased melanoma risk [13]. There is evidence from the United Kingdom and elsewhere that TSSE practice in people with melanoma is suboptimal and not practiced monthly as recommended [14,15]. Barriers to initiating and maintaining TSSE include lack of initial training, declining motivation, and insufficient time [16]. There are good reasons to believe that these barriers could be tackled by digital technology [15]. However, it is also important to ensure that interventions to increase TSSE do not have the unintended consequence of negatively affecting patient well-being. It has been shown that long-term survivors of cancer have increased rates of anxiety compared with controls [17]. Furthermore, there is evidence of increasing anxiety in the days and weeks preceding a scheduled follow-up appointment for many survivors of melanoma [18]. As such, it is possible that more frequent prompts to check the skin between scheduled follow-ups will exacerbate patient anxiety and adversely affect well-being.

### This Study

The aim of this pilot study was to evaluate the Achieving Self-directed Integrated Cancer Aftercare (ASICA) self-directed digital intervention in a patient-focused randomized controlled trial among those treated for a first stage 0 to IIC primary cutaneous melanoma within the preceding 60 months. The primary objective of the pilot study was to determine the impact of using ASICA on patients’ melanoma worry, anxiety and depression, and quality of life. The secondary objective was to provide information on the feasibility of the processes for a full-scale national trial of the ASICA intervention.

## Methods

### Study Protocol

The trial protocol has been published and is available as a web-based supplement [19]. The methods are described briefly in the following sections according to the CONSORT (Consolidated Standards of Reporting Trials) guidelines.

### Study Design and Participants

**Figure 1:**
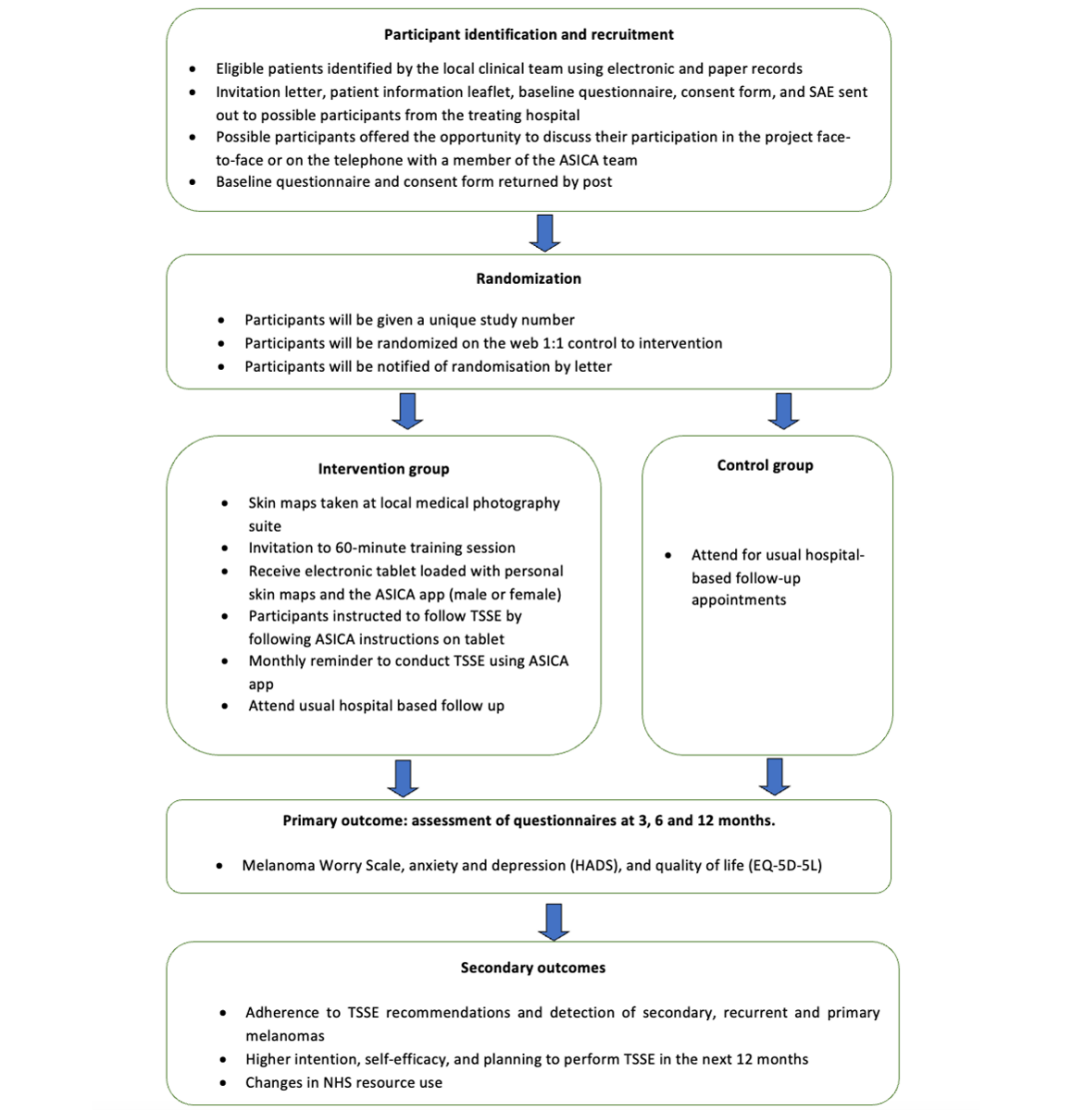
Flow diagram of the study design and schedule. Reproduced from Murchie et al [20]. This paper is distributed under the terms of the Creative Commons Attribution 4.0 International License, which permits unrestricted use, distribution, and reproduction in any medium provided appropriate credit is given to the original authors and source, a link to the Creative Commons license is provided, and it is indicated if changes were made. The Creative Commons Public Domain Dedication waiver [30] applies to the data made available in this paper unless otherwise stated. ASICA: Achieving Self-directed Integrated Cancer Aftercare; HADS: Hospital Anxiety and Depression Scale; NHS: National Health Service; SAE: self-addressed envelope; TSSE: total skin self-examination.

ASICA was a 2-arm, open, 2-center randomized controlled pilot trial comparing the ASICA digital intervention with a control group receiving usual follow-up only (Figure 1 [20,21]). The study sites were the Aberdeen Royal Infirmary and Addenbrooke’s Hospital, Cambridge. Adults (aged ≥18 years) treated within the preceding 60 months for a previous stage 0 to IIC primary cutaneous melanoma were sent information about the study, a consent form, and a baseline questionnaire by post. Individuals diagnosed with stage III and IV melanoma or recurrent melanoma within the last 60 months or unable to consent or complete the questionnaires were excluded. Those interested in participating in the study were contacted by the recruiting site for further discussion. The participants were randomized after informed written consent had been obtained.

### Intervention and Control

The intervention group received the ASICA digital tablet-based intervention, which is designed to support TSSE in those with cutaneous melanoma and enables appropriate and timely clinical responses when concerns are raised. It has been rigorously developed and is theoretically based, using specified behavior change techniques to prompt users to perform regular TSSE [20].

Briefly, the intervention group participants attended a 30-minute training session in which they were provided with a 7-inch Samsung Galaxy tablet and given instructions on the intervention and how the tablet-based app should be used to support them in conducting a thorough, full-body TSSE in response to a monthly SMS text message reminder sent from the trial team. The nurse demonstrated the function of the app and answered any questions about the TSSE or the intervention. The app included information about the importance of monthly TSSE; instructional videos demonstrating how to perform a TSSE and take good photographs of skin lesions; a digital map of the patient’s own skin; a structured checkbox list of body parts to check; prompts for the patient to plan their next TSSE; and the capability to take photographs of suspicious skin lesions and send them to a dermatology nurse practitioner for review along with a text-based report of the TSSE outcomes, including a description of any concerns. All participants who submitted text-based reports of any skin concerns were followed up with by the dermatology nurse practitioner*.* The monthly prompt was sent on a single occasion, and no reminders were sent to individuals who did not complete the TSSE that month, but they would continue to be reminded on each subsequent month. The control group also completed the baseline questionnaire. All participants (intervention and control) continued to attend their usual structured melanoma follow-up as determined by local guidelines.

### Randomization and Blinding

Participants were randomized 1:1 to intervention or control using a remote automated computer-allocated application hosted at the Centre for Healthcare Randomised Trials in Aberdeen, United Kingdom. An algorithm minimized the imbalance in sex and center between the groups [22]. Owing to the nature of the intervention, both participants and researchers were not blinded to the randomized allocation.

### Outcomes and Ascertainment

Baseline data were collected from secondary care records by a research nurse at each site before randomization. The coprimary outcomes were the Melanoma Worry Scale, anxiety and depression (Hospital Anxiety and Depression Scale), and quality of life (EQ-5D-5L) [19]. The secondary outcomes were adherence to TSSE recommendations, self-efficacy, and future intention and planning to perform TSSE [23]. Primary and secondary outcomes were collected using postal questionnaires at baseline and 3, 6, and 12 months after randomization. Tertiary outcomes were new primary and recurrent melanomas and patterns of skin-related NHS resource use. These were collected 12 months after randomization from secondary care records by research nurses blinded to allocation.

### Sample Size

There was no formal power calculation to derive sample size. The decision to conduct a relatively large pilot randomized controlled trial of 240 participants was influenced by several factors. Our previous nonrandomized feasibility study recruited 19 patients to provide information on recruitment, acceptability, compliance, and retention at 1 site [19]. Hospital Anxiety and Depression Scale scores at the 6-month follow-up exhibited high variability in both magnitude and direction of the effect at follow-up. This raised the possibility of a bidirectional effect on psychological outcomes (ie, some individuals were made more and some less anxious by the intervention). Another possible explanation was, of course, a small, unrepresentative sample. This required further exploration in a sample of sufficient size and representativeness before proceeding to a trial powered on clinical outcomes. A sample size of 240 was a pragmatic choice to provide a sufficiently diverse group of participants (with respect to age, sex, geographical location, and socioeconomic status) to assess this.

### Statistical Analysis

A comprehensive statistical analysis plan was agreed upon with the trial steering committee before any analysis and is available upon request from the corresponding author. The analysis was based on the intention-to-treat principle. No interim analyses were planned or conducted. Baseline characteristics and follow-up data were described using summary statistics (mean and SD or median and IQR for continuous variables dependent on distribution and number and percentage for categorical variables). Treatment effects are presented with 95% CIs. There were no adjustments to the secondary outcome CIs for multiple testing.

A linear mixed effects, repeated-measure model was used for the analysis of the coprimary outcomes. The treatment group (ASICA or control), time point (3, 6, and 12 months), trial center (Aberdeen or Cambridge), and baseline value for the outcome variable were included as fixed effects. A treatment-by-time interaction was included to estimate the treatment effect at each time point. A random effect was included for participants. Other covariates in the model were age and time since diagnosis (years) as continuous variables and fixed effects for sex, deprivation (decile), rurality (urban vs rural), site (head and neck, upper body, upper limb, and lower limbs), and stage (0, IA, IB, and II) of melanoma at baseline as categorical variables.

TSSE question scores were aggregated to obtain domain scores for intentions, self-efficacy and planning to conduct TSSE. TSSE practice at 12 months was compared between the groups by calculating the difference in proportions with 95% CIs with continuity correction between trial groups. A stringent definition of TSSE practice as described by Janda et al [23] was used as an outcome compared between the trial groups. For a participant to be considered to have performed a TSSE, they must also report that they used a mirror or asked for help from someone else to examine difficult-to-see areas of their skin. A logistic regression model was then used to analyze this, adjusted for similarly defined baseline TSSE. The difference in mean scores between the groups was estimated for TSSE self-efficacy, intention, and planning using analysis of covariance controlling for baseline values of these same outcomes (TSSE self-efficacy, intention, and planning).

Negative binomial regression was used to estimate the incidence rate ratios (IRRs) of the ASICA group compared with the control group with respect to the use of resources, as evidenced by skin-related general practitioner (GP) appointments, hospital appointments, and hospital admissions. The models were adjusted for baseline age, sex, deprivation, rurality, time since diagnosis, site, and stage of melanoma. A negative binomial regression model was also used for intention to conduct TSSE at the 12-month follow-up (the number of times the patient planned to conduct TSSE in the following 12 months) controlling for baseline intentions.

### Ethical Considerations

This project received full approval from the North of Scotland Research Ethics Committee on April 28, 2017 (17/NS/0040). Written informed consent was obtained from all study participants. The trial was conducted according to the principles of good clinical practice provided by the Research Governance Guidelines. Consent for publication did not apply.

### Patient and Public Involvement

A detailed pilot study was conducted during the development of the ASICA project to ascertain patients’ priorities, experiences, and preferences. Interviews were conducted with 19 potential recipients of the ASICA intervention, and these interviews informed the development of the study research questions and the selection of outcome measures. Patients were not directly involved in the design of the study but did inform the design via participation in the pilot study interviews. The burden of the ASICA intervention was assessed by patients in a qualitative substudy. A total of 2 patient representatives sat on the trial steering committee feeding into plans for recruitment and dissemination. The results of the project will be disseminated to all participants (except for those who opted out) via a postal newsletter.

### Trial Status

Participant recruitment began in January 2018 and finished in March 2019. The first participant was randomized on January 24, 2018. Currently approved protocol: version 3, May 1, 2020.

## Results

### Overview

**Figure 2:**
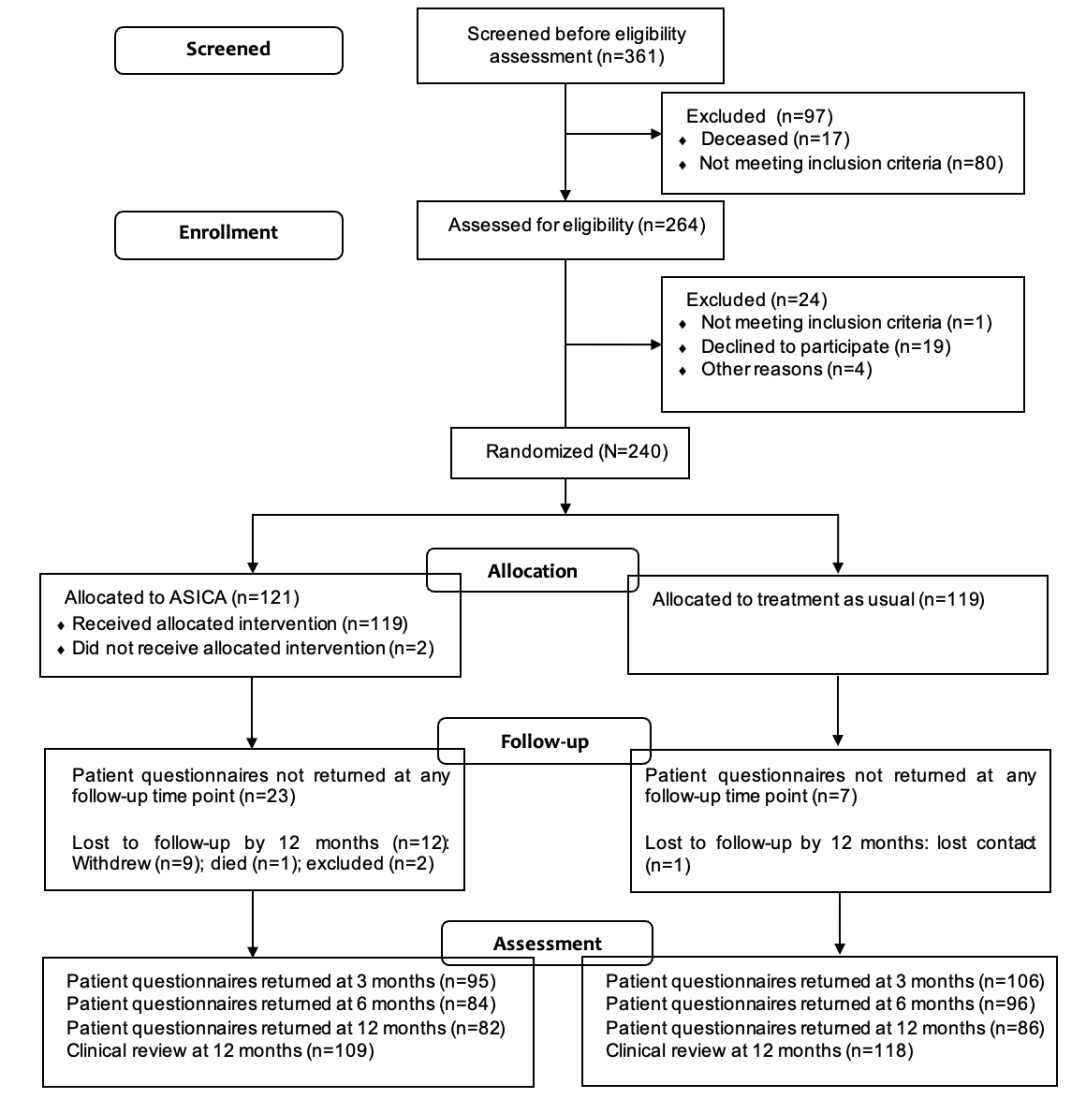
Flow diagram of participants through the Achieving Self-directed Integrated Cancer Aftercare (ASICA) trial.

Between January 24, 2018, and March 8, 2019, a total of 240 participants were randomized (n=121, 50.4% to the ASICA intervention and n=119, 49.6% to usual care). A total of 264 participants from the 2 centers were assessed for eligibility for the trial (n=188, 71.2% at the Aberdeen Royal Infirmary and n=76, 28.8% at the Addenbrooke’s Hospital, Cambridge). Of these 264 participants, 19 (7.2%) declined participation, 1 (0.4%) did not meet the inclusion criteria, and 4 (1.5%) had other reasons. At 12 months, 67.8% (82/121) of the participants in the ASICA group returned patient questionnaires, whereas 72.3% (86/119) of the participants in the usual follow-up group returned completed questionnaires (Figure 2). The baseline demographic and clinical characteristics were balanced between the 2 trial groups (Table 1).

**Table 1:** Baseline demographic and clinical characteristics and outcome measures for the trial participants (N=240).

			ASICA^a^ (n=121)	Control group (n=119)
**Characteristics**				
	Sex (male), n (%)		55 (45.5)	53 (44.5)
	Age (years), mean (SD)		59.1 (14.1)	57.6 (13.7)
	Time since diagnosis (years), mean (SD)		2 (1.3)	1.9 (1.3)
	**Deprivation decile, n (%)**			
		1 (most deprived)	0 (0)^b^	0 (0)^c^
		2	2 (1.7)^b^	2 (1.7)^c^
		3	4 (3.4)^b^	3 (2.5)^c^
		4	0 (0)^b^	7 (5.9)^c^
		5	4 (3.4)^b^	9 (7.6)^c^
		6	17 (14.5)^b^	12 (10.2)^c^
		7	16 (13.7)^b^	13 (11)^c^
		8	18 (15.4)^b^	20 (16.9)^c^
		9	28 (23.9)^b^	22 (18.6)^c^
		10 (least deprived)	28 (23.9)^b^	30 (25.4)^c^
	**Rurality, n (%)**			
		Urban	72 (59.5)	78 (65.5)
		Rural	49 (40.5)	41 (34.5)
**Clinical characteristics**				
	**Site of first primary melanoma, n (%)**			
		Head and neck	22 (18.2)	22 (18.5)
		Upper body	46 (38)	51 (42.9)
		Upper limbs	21 (17.4)	21 (17.6)
		Lower limbs	32 (26.4)	25 (21)
	**Subtype of melanoma at diagnosis, n (%)**			
		Superficial spreading	86 (72.3)^d^	88 (75.9)^e^
		Nodular	10 (8.4)^d^	3 (2.6)^e^
		Amelanotic	1 (0.8)^d^	0 (0)^e^
		Lentigo maligna	8 (6.7)^d^	13 (11.2)^e^
		Acral	1 (0.8)^d^	1 (0.9)^e^
		Other	13 (10.9)^d^	11 (9.5)^e^
	**Stage of melanoma diagnosis, n (%)**			
		0 and IA	57 (47.1)	65 (54.6)
		IB	43 (35.5)	39 (32.8)
		IIA, IIB, and IIC	21 (17.4)	15 (12.6)
	Breslow depth (mm), median (IQR)		0.9 (0.5-1.6)	0.6 (0.5-1.1)
	**Clark level, n (%)**			
		1	0 (0)^f^	0 (0)^g^
		2	15 (31.9)^f^	13 (29.5)^g^
		3	10 (21.3)^f^	12 (27.3)^g^
		4	21 (44.7)^f^	19 (43.2)^g^
		5	1 (2.1)^f^	0 (0)^g^
	**Mode of detection, n (%)**			
		Patient-detected	27 (69.2)^h^	36 (100)^i^
		Detected at hospital	5 (12.8)^h^	0 (0)^i^
		Detected by GP^j^	1 (2.6)^h^	0 (0)^i^
		Other	6 (15.4)^h^	0 (0)^i^
	**Type of melanoma treatment, n (%)**			
		Surgery	120 (99.2)	118 (99.2)
		Immunotherapy	0 (0)	0 (0)
		Radiotherapy	1 (0.8)	0 (0)
		Chemotherapy	1 (0.8)	0 (0)
**Outcome measures**				
	Melanoma Worry Scale, mean (SD)		8.5 (3.5)^k^	8.8 (3.1)^e^
	**HADS^l^, mean (SD)**			
		Anxiety	5 (4.1)^c^	5.1 (3.5)^b^
		Depression	2.8 (2.9)^c^	2.8 (2.6)^e^
	Quality of life (EQ-5D-5L), mean (SD)		0.871 (0.148)^b^	0.863 (0.158)^b^
	**Resource use in preceding 2 years, median (IQR)^m^**			
		Melanoma follow-up appointments	4 (2-5.3)	3 (2-5)
		Skin-related hospital appointments	2 (1-3)	1 (1-2)
		Skin-related hospital admissions	1 (1-2)	1.5 (1-2)
	Reported practicing TSSE^n^ in previous 12 months, n (%)		60 (63.2)^o^	73 (74.5)^p^

^a^ASICA: Achieving Self-directed Integrated Cancer Aftercare.

^b^N=117.

^c^N=118.

^d^N=119.

^e^N=116.

^f^N=47.

^g^N=44.

^h^N=39.

^i^N=36.

^j^GP: general practitioner.

^k^N=115.

^l^HADS: Hospital Anxiety and Depression Scale.

^m^Of those who used these resources.

^n^TSSE: total skin self-examination (defined as having used a mirror or asked for help to view difficult-to-see areas of the skin).

^o^N=95.

^p^N=98.

### Melanoma Worry

The difference between the groups for melanoma worry score was close to 0 at all time points (Table 2), and the narrow CI bands indicated that ASICA did not increase melanoma worry among the intervention group at any point at which it was measured during the trial.

**Table 2:** Estimates for mean differences at each time point for the primary outcomes (N=240).

Outcome, subscale, and time point			ASICA^a^ (n=121), mean (SD)	Control group (n=119), mean (SD)	Adjusted mean difference^b^ (95% CI)	*P* value
**MWS^c^**						
	3 months		8.47 (3.03)^d^	8.48 (2.93)^e^	0.23 (–0.31 to 0.78)	.40
	6 months		7.65 (2.71)^f^	7.97 (3.13)^g^	–0.1 (–0.70 to 0.51)	.76
	12 months		7.94 (3.20)^f^	7.93 (3.06)^h^	0.12 (–0.60 to 0.84)	.74
**HADS^i^**						
	**Anxiety**					
		3 months	4.17 (3.6)^j^	4.57 (3.78)^d^	–0.13 (–0.79 to 0.54)	.71
		6 months	3.55 (3.25)^k^	4.71 (4.28)^l^	–1.00 (–1.74 to –0.26)	.01
		12 months	3.77 (3.41)^k^	4.38 (3.95)^m^	–0.54 (–1.31 to 0.23)	.17
	**Depression**					
		3 months	2.33 (2.35)^l^	2.79 (3.19)^n^	–0.24 (–0.84 to 0.35)	.42
		6 months	2.05 (2.43)^k^	3.18 (3.35)^j^	–0.77 (–1.41 to –0.12)	<.001
		12 months	2.28 (2.69)^o^	2.82 (3.35)^p^	–0.44 (–1.11 to 0.23)	.20
**Quality of life (EQ-5D-** **5L** **)**						
	3 months		0.877 (0.137)^q^	0.864 (0.169)^e^	0.024 (–0.006 to 0.054)	.11
	6 months		0.911 (0.129)^r^	0.853 (0.19)^d^	0.070 (0.032 to 0.107)	<.001
	12 months		0.891 (0.136)^f^	0.859 (0.177)^h^	0.044 (0.003 to 0.085)	.04

^a^ASICA: Achieving Self-directed Integrated Cancer Aftercare.

^b^Adjusted for baseline scores, age, sex, deprivation, rurality, time since diagnosis, site, and stage of melanoma.

^c^MWS: Melanoma Worry Scale.

^d^N=92.

^e^N=102.

^f^N=80.

^g^N=93.

^h^N=84.

^i^HADS: Hospital Anxiety and Depression Scale.

^j^N=90.

^k^N=75.

^l^N=89.

^m^N=73.

^n^N=95.

^o^N=76.

^p^N=77.

^q^N=94.

^r^N=83.

### Anxiety and Depression

The ASICA group had lower anxiety scores at each time point compared with the control group, but these differences were small, and CIs showed that larger differences were not compatible with the data (Table 2). At 12 months, the difference was –0.54 (95% CI –1.31 to 0.23; *P*=.17). This pattern was similar for depression; at 12 months, the mean difference was –0.44 (95% CI –1.11 to 0.23; *P*=.20).

### Quality of Life

The EQ-5D-5L also favored ASICA at each time point (Table 2). At 12 months, it was higher in the ASICA group, with a mean difference of 0.044 (95% CI 0.003-0.085; *P*=.04).

### Secondary Outcomes

#### Self-reported TSSE Adherence

Table 3 reports between-group comparisons of secondary outcomes of any TSSE practice, resource use, TSSE intentions, TSSE self-efficacy, and TSSE planning during the study year. Table 4 provides more details from questionnaire responses about self-reported TSSE practice during the study year.

**Table 3:** Estimates for secondary outcomes at the 12-month follow-up.

Outcome and subcategory				ASICA^a^		Control group		Effect estimates (95% CI)		*P* value
Self-reported TSSE^b^ at 12 months^c^—Yes, n (%)				58 (76)^d^		47 (73)^e^		2.45 (0.76 to 7.90)		.13
**Resource use, median (IQR); mean (SD)**										
	Skin-related GP^f^ appointments^g^			0 (0-0); 0.27 (0.79)^h^		0 (0-0); 0.13 (0.46)^i^		2.64 (1.1 to 6.33)		.03
	Skin-related hospital appointments^g^			0 (0-1); 0.66 (1.35)^j^		0 (0-1); 0.49 (0.95)^k^		1.14 (0.71 to 1.85)		.59
	Skin-related hospital admissions^g^			0 (0-1); 0.53 (0.92)^l^		0 (0-0); 0.28 (0.58)^m^		1.94 (1.17 to 3.2)		.01
**TSSE,** **mean (SD)**										
	Intentions about TSSE^n^			11.9 (8.9)^o^		8.3 (14.5)^p^		1.44 (0.97 to 2.13)		.07
	Self-efficacy about TSSE^q^			33.5 (6.0)^r^		29.9 (6.9)^s^		3.8 (2.0 to 5.6)		<.001
	**Planning about TSSE**									
		Action planning	7.3 (2.1)^t^		5.9 (2.2)^u^		1.3 (0.6 to 1.1)		<.001	
		Coping planning	4.22 (0.77)^t^		3.96 (0.79)^v^		0.24 (–0.01 to 0.50)		.06	

^a^ASICA: Achieving Self-directed Integrated Cancer Aftercare.

^b^TSSE: total skin self-examination.

^c^Self-reported TSSE defined as having used a mirror or asked for help to view difficult-to-see areas of the skin. The effect estimate is the odds ratio adjusted for baseline self-reported TSSE.

^d^N=76.

^e^N=64.

^f^GP: general practitioner.

^g^The effect estimates are incidence rate ratios adjusted for center, age at randomization, sex, deprivation decile, rurality, time since diagnosis, site of melanoma, and stage of melanoma.

^h^N=82.

^i^N=86.

^j^N=92.

^k^N=91.

^l^N=89.

^m^N=90.

^n^The effect estimate is the incidence rate ratio adjusted for baseline intentions.

^o^N=56.

^p^N=55.

^q^The effect estimates are the differences in means adjusted for the baseline outcome score.

^r^N=74.

^s^N=72.

^t^N=73.

^u^N=70.

^v^N=67.

**Table 4:** Total skin self-examination practice at 12 months.

Question	ASICA^a^, n (%)	Control group, n (%)	Difference in proportion (95% CI)	*P* value
Have you or someone who is not a doctor or nurse, such as your spouse or partner, ever deliberately checked any part of your skin for early signs of skin cancer?—Yes	64 (88)^b^	62 (82)^c^	6.1 (–6.8 to 19.0)	.42
In the past 12 months, have you or someone who is not a doctor or nurse, such as your spouse or partner, deliberately checked any part of your skin for early signs of skin cancer?—Yes	63 (95)^d^	58 (89)^e^	6.3 (–4.4 to 16.8)	.31
In the past 12 months, how often have you or someone who is not a doctor or nurse checked any part of your skin for early signs of skin cancer?—≥5 times	45 (68)^d^	25 (42)^f^	26.5 (8.1 to 44.9)	.005
And just thinking about the past 6 months, how often have you or someone who is not a doctor or nurse checked any part of your skin for early signs of skin cancer?—≥5 times	35 (53)^d^	17 (29)^g^	24.2 (5.9 to 42.5)	.01
During your last check, did you use a handheld mirror or full-sized mirror to check difficult-to-see areas of your skin such as your back?—Yes	50 (74)^h^	31 (48)^i^	25.1 (9.0 to 41.2)	.005
During your last check, did you have someone to help you see difficult-to-see areas; for example, your wife, partner, or another relative?—Yes	36 (53)^h^	38 (60)^j^	–7.4 (–25.8 to 11.1)	.50

^a^ASICA: Achieving Self-directed Integrated Cancer Aftercare.

^b^N=73.

^c^N=76.

^d^N=66.

^e^N=65.

^f^N=60.

^g^N=59.

^h^N=68.

^i^N=64.

^j^N=63.

A higher proportion of the ASICA group (58/76, 76%) than of the control group (47/64, 73%) reported having conducted at least one TSSE during the study year, but the difference was nonsignificant (*P*=.13). However, a significantly higher proportion of the ASICA group reported checking their skin 5 or more times over the 12 months of follow-up compared with the control group (45/66, 68% vs 25/60, 42%; between-group difference: 26.5, 95% CI 8.1-44.9; *P*=.005). A significantly greater proportion in the ASICA group than in the control group reported having used a mirror to check difficult-to-see areas of their skin (50/68, 74% vs 31/64, 48%; between-group difference: 25.1, 95% CI 9.0-41.2; *P*=.005). Details of the difference in the proportion of actual TSSE practice at 12 months are reported in Table 4. When using the stringent TSSE practice definition, there were higher but nonsignificant odds of reporting having carried out TSSE in the ASICA arm than in the usual follow-up arm (odds ratio 2.45, 95% CI 0.76-7.90; *P*=.13) allowing for baseline self-reported TSSE.

#### Intention, Self-efficacy, and Planning to Conduct TSSE

Table 3 reports the effect estimates for participants’ intentions, self-efficacy, and planning to conduct TSSE. Participants’ intentions to check their skin for early signs of cancer were similar in the 2 groups, as were the intentions to contact a health professional if they found something of concern during TSSE. Participants in the ASICA group reported having a significantly higher level of confidence (self-efficacy) about checking their skin correctly than the usual care group (mean difference: 3.8, 95% CI 2.0-5.6; *P*<.001). The ASICA group also had clearer plans about when and where they would conduct TSSE (action planning; mean difference: 1.3, 95% CI 0.6-1.1; *P*<.001).

#### Patterns of NHS Resource Use

The rate of skin-related GP appointments reported by participants was significantly higher in the ASICA group than in the control group (adjusted IRR: 2.64, 95% CI 1.1-6.33; *P*=.03). In addition, the rate of melanoma-related hospital admissions was higher in the ASICA group than in the control group (IRR: 1.94, 95% CI 1.17-3.2; *P*=.01); however, there was no difference in the rate of skin-related hospital appointments between the groups (IRR: 1.14, 95% CI 0.71-1.85; *P*=.59).

#### Recurrences and New Primaries

There were 4.1% (5/121) of recurrences or new primaries reported in the ASICA group compared with 9.2% (11/119) in the control group (odds ratio 0.42, 95% CI 0.14-1.26; *P*=.18).

## Discussion

### Summary of Principal Findings

This pilot study succeeded in recruiting 241 survivors of melanoma. Overall, the results demonstrate that ASICA is a feasible and acceptable means of supporting TSSE practice in survivors of melanoma. In the pilot study, using ASICA did not increase melanoma worry and led to a significant reduction in anxiety and depression scores at 6 months but not at 12 months. ASICA users reported a significantly higher quality of life at 6 and 12 months. These results provide an important signal suggesting that widespread ASICA use by survivors of melanoma would have no adverse psychological effects and may improve quality of life. Furthermore, during the study year, ASICA users reported checking their skin more frequently and thoroughly than the control participants. ASICA users also reported that they were more confident in their ability to check their skin and had clearer plans regarding when and where they would perform the checks. Furthermore, ASICA users had significantly more skin-related GP appointments and hospital admissions.

### Strengths and Limitations

This study is timely given the growing interest in and research activity on digital health care interventions in modern health services. Good quality evidence to inform policy and best practices in the field is needed. Our trial implemented and evaluated a rigorously developed and theoretically based digital intervention with real potential to improve patient outcomes and the efficiency of services. The trial was sufficiently large to provide strong signals about the likely impact of using the ASICA intervention on participants’ psychological well-being and quality of life, although a larger trial with a sample size calculation informed by these results will be needed to provide definitive evidence of psychological benefit. Furthermore, the trial was designed to capture how well potential recipients of a digital intervention actually used it. The trial also measured the psychological variables (self-efficacy, intention, and planning) that are most predictive of continuing behavior change [19].

The trial has informed on the overall feasibility of ASICA being used by survivors of melanoma. It has also provided useful information about trial procedures and crucially enabled insight into practical issues relating to the use of ASICA from the perspective of the different population groups that could take part in a definitive trial powered on clinical outcomes and among whom the intervention would ultimately be implemented. The use of the 2 study sites has provided confidence that individuals in remote locations can be monitored successfully by an appropriately skilled dermatology nurse practitioner.

Less affluent individuals were underrepresented in the participants. In some ways, this reflects the demographic profile of melanoma in the United Kingdom and, therefore, the likely future users of ASICA. By contrast, it emphasizes that it is challenging to recruit those of lower economic status to clinical trials, with the resultant effect of increasing “health data poverty” regarding how those with lower economic status engage with technology to manage their health [24]. Specifically, in this trial, it means that we lack definitive detailed knowledge of how effectively deprived individuals could or would use the ASICA intervention, which may hinder future optimization of the intervention and its wider implementation. However, it may be that future development of ASICA could include a web-accessible demonstration that might be disseminated using social media, and this could enable us to reach groups that are harder to recruit to trials using traditional recruitment mechanisms [25]. However, this is an important point and emphasizes the importance of considering methods to increase demographic equity of recruitment in digital health care trials going forward [25]. A further point to note is that there were differing degrees of adherence to the intervention displayed by the intervention group. Although adherence was not a prespecified outcome for this study, data on adherence patterns were collected and will be reported separately.

ASICA represents a complex intervention consisting of 3 interconnecting components: a prompt to conduct a TSSE, an app to support the conduction and reporting of a monthly TSSE, and a clinical response where concerns were raised. The challenges of evaluating complex interventions and of being certain of how the complex components have achieved any observed effects are well described. To provide the best opportunity to understand how our intervention worked, we first developed it carefully and sequentially with potential users in a series of developmental steps [19]. Second, we measured our primary and secondary outcomes using established and validated instruments [20]. Third, we conducted parallel qualitative interviews with a sample of participants to obtain a clearer understanding of how ASICA operates in the field. These data are beyond the scope of this paper but will be reported separately. However, there remains the challenge inherent in all evaluations of being certain of how intervention components have operated together to produce the apparent effects reported in this paper.

### Context With Other Literature

Evidence for the place of digital technology to support those at high risk of melanoma as well as survivors of melanoma is accumulating. A trial in the East of England randomized 119 of 238 people at high risk of melanoma to use a smartphone skin self-monitoring app for 12 months. The study found no increase in skin self-monitoring behavior or skin consultation in the intervention group but, equally, found no evidence of increased melanoma worry. This adds to our finding that digitally supported skin self-monitoring is not psychologically harmful [26]. ASICA users also reported having checked their skin more regularly and thoroughly during the study year, and this seems to have resulted in a greater number of subsequent GP appointments and skin-related hospital admissions. This is consistent with an earlier study in which recipients of an educational program to increase TSSE were found to have increased rates of skin surgery [13]. It could be that increased TSSE practice does make individuals more vigilant and more inclined to seek medical advice for concerning skin lesions, with a corresponding increase in biopsies to establish a definitive diagnosis.

A possible limitation of the ASICA intervention is that it is relatively “low-tech” and does not use the latest technologies, such as teledermoscopy or artificial intelligence. A study in Queensland, Australia, randomized half of 234 participants with at least two risk factors for melanoma to use a smartphone-based dermatoscope for skin self-monitoring, with the control being naked-eye skin self-monitoring for 2 months. Mobile teledermoscopy did not increase sensitivity for detection of skin cancers [27]. In terms of artificial intelligence, a recent review including 9 studies of 6 different algorithm-based smartphone apps concluded that the apps could not be relied upon to detect melanoma or other skin cancers. The reviewers suggested that test performance is likely to be poorer than reported if the apps are used in clinically relevant populations and by their intended users [28]. In light of the data presented here, it appears that our approach has the potential to offer efficient and effective digital survivorship care for patients with melanoma in the short to medium term.

Adding human support is also known to promote engagement in many interventions [29]. A key feature of our intervention compared with similar interventions for skin cancer was that it enabled participants who had raised concerns to interact via telephone and the internet to receive support and guidance from a dermatology nurse practitioner. The beneficial role of a human guide in promoting engagement with digital interventions has been noted previously; for example, by a systematic review of 14 studies of internet-based mental health interventions [30].

### Conclusions and Implications

Using ASICA did not worsen psychological well-being and appeared to reduce anxiety and depression and improve quality of life in this demographically diverse group of survivors of melanoma. ASICA users also reported performing more regular TSSE and having greater confidence in conducting and planning it. Overall, these findings reinforce the potential for ASICA to support survivors of melanoma in the future. Further work could focus on incorporating elements of artificial intelligence and automation to increase efficiency and improve adherence [29].

## Appendix

Multimedia Appendix 1 CONSORT eHEALTH Checklist (V 1.6.2).

## Abbreviations

ASICA: Achieving Self-directed Integrated Cancer Aftercare
CONSORT: Consolidated Standards of Reporting Trials
GP: general practitioner
IRR: incidence rate ratio
NHS: National Health Service
TSSE: total skin self-examination

## Data Availability

The intellectual property of the Achieving Self-directed Integrated Cancer Aftercare intervention is held by the University of Aberdeen. Regulatory approval and ethical approval received from the participants makes it possible for data collected during this study (including anonymized individual patient data) and a data dictionary defining each field within the data set to be made available to other bona fide researchers subject to regulatory approvals and the agreement of the principal investigator (PM). Other related documents (including the study protocol, statistical analysis plan, study questionnaires, case report forms, consent forms, and invitation letters) are available from the corresponding author upon request. Data will be made available from publications through application to the corresponding author. The participants have been assured that no data that could personally identify them will be shared in any circumstance, and that any data shared will be the minimum required to address the needs of the applicant. Data-sharing requests should be made to the principal investigator and will be granted only upon careful consideration of the specific case made for why the data are to be shared, how they are to be used, and the likely outcome of their use. Decisions to share data will only be made in light of advice from the regulatory bodies approving the study and from Research and Innovation, University of Aberdeen. Where a decision is made to share data, we will ensure that secure transfer protocols are used and that optimal security is used by the data recipient. Upon application to the corresponding author, we will also provide copies of the study protocol, statistical analysis plan, and other relevant study documentation such as informed consent forms. Data are available for sharing from June 23, 2021. The data will be made available by the corresponding author (PM).
