# Supplementary material for: The Achieving Self-directed Integrated Cancer Aftercare Intervention for Detection of Recurrent and Second Primary Melanoma in Survivors of Melanoma: Pilot Randomized Controlled Trial
Source: JMIR Cancer. 2022 Sep 8;8(3):e37539. doi: 10.2196/37539 (PMC9501683; doi:10.2196/37539)
Supplement: Multimedia Appendix 1 [file cancer_v8i3e37539_app1.pdf]

CONSORT-EHEALTH Checklist V1.6.2 Report	Manuscript Number	37539

(based on CONSORT-EHEALTH V1.6), available at [http://tinyurl.com/consort-ehealth-v1-6].

Date completed

9/7/2022 13:09:47

by

Peter Murchie

The Achieving Self-directed Integrated Cancer Aftercare Intervention for Detection of Recurrent and Second Primary Melanoma in Survivors of Melanoma: Pilot Randomized Controlled Tria

TITLE

1a-i) Identify the mode of delivery in the title

The Achieving Self-Directed Integrated Cancer Aftercare (ASICA) intervention for detection of recurrent and second primary melanoma in melanoma survivors: A randomised controlled pilot trial

1a-ii) Non-web-based components or important co-interventions in title

The Achieving Self-Directed Integrated Cancer Aftercare (ASICA) intervention for detection of recurrent and second primary melanoma in melanoma survivors: A randomised controlled pilot trial

1a-iii) Primary condition or target group in the title

The Achieving Self-Directed Integrated Cancer Aftercare (ASICA) intervention for detection of recurrent and second primary melanoma in melanoma survivors: A randomised controlled pilot trial

ABSTRACT

1b-i) Key features/functionalities/components of the intervention and comparator in the METHODS section of the ABSTRACT

ASICA (Achieving Self-directed Integrated Cancer Aftercare) a tablet-based digital intervention to prompt and support TSSE in melanoma survivors, or to usual care

1b-ii) Level of human involvement in the METHODS section of the ABSTRACT

Adults (aged ≥18) diagnosed with a first 0-IIC primary cutaneous melanoma were randomised to receive ASICA (Achieving Self-directed Integrated Cancer Aftercare) a tablet-based digital intervention to prompt and support TSSE in melanoma survivors, or to usual care.

1b-iii) Open vs. closed, web-based (self-assessment) vs. face-to-face assessments in the METHODS section of the ABSTRACT

Adults (aged ≥18) diagnosed with a first 0-IIC primary cutaneous melanoma were randomised to receive ASICA (Achieving Self-directed Integrated Cancer Aftercare) a tablet-based digital intervention to prompt and support TSSE in melanoma survivors, or to usual care.

1b-iv) RESULTS section in abstract must contain use data

Adults (aged ≥18) diagnosed with a first 0-IIC primary cutaneous melanoma were randomised to receive ASICA (Achieving Self-directed Integrated Cancer Aftercare) a tablet-based digital intervention to prompt and support TSSE in melanoma survivors, or to usual care.

1b-v) CONCLUSIONS/DISCUSSION in abstract for negative trials

Using ASICA for 12 months does not increase melanoma worry, can reduce anxiety and depression and may improve quality of life. ASICA has the potential to improve well-being and vigilance of melanoma survivors and enable the benefits of regular TSSE

INTRODUCTION

2a-i) Problem and the type of system/solution

The aim of this pilot study was to evaluate the ASICA self-directed digital intervention in a patient-focused randomized controlled trial among those treated for a first stage 0-2C primary cutaneous melanoma within the preceding 60 months. The primary objective of the pilot study was to determine the impact of using ASICA on patient’s melanoma worry, anxiety and depression and quality of life. The secondary objective was to provide information on the feasibility of processes for a full-scale national trial of the ASICA intervention

2a-ii) Scientific background, rationale: What is known about the (type of) system

The aim of this pilot study was to evaluate the ASICA self-directed digital intervention in a patient-focused randomized controlled trial among those treated for a first stage 0-2C primary cutaneous melanoma within the preceding 60 months. The primary objective of the pilot study was to determine the impact of using ASICA on patient’s melanoma worry, anxiety and depression and quality of life. The secondary objective was to provide information on the feasibility of processes for a full-scale national trial of the ASICA intervention

Does your paper address CONSORT subitem 2b?

The aim of this pilot study was to evaluate the ASICA self-directed digital intervention in a patient-focused randomized controlled trial among those treated for a first stage 0-2C primary cutaneous melanoma within the preceding 60 months. The primary objective of the pilot study was to determine the impact of using ASICA on patient’s melanoma worry, anxiety and depression and quality of life. The secondary objective was to provide information on the feasibility of processes for a full-scale national trial of the ASICA intervention

METHODS

3a) CONSORT: Description of trial design (such as parallel, factorial) including allocation ratio

ASICA was a two-arm, open, two-centre randomised controlled pilot trial (RCT) comparing the ASICA digital intervention with a control group receiving usual follow-up only (Figure 1). The study sites were Aberdeen Royal Infirmary and Addenbrooke’s Hospital, Cambridge. Adults (≥18 years) treated within the preceding 60 months for a previous stage 0-IIC primary cutaneous melanoma were sent information about the study, a consent form and baseline questionnaire by post. Individuals diagnosed with stage 3 and 4 melanoma, recurrent melanoma within the last 60 months or unable to consent and/or complete questionnaires were excluded. Those interested in participating in the study were contacted by the recruiting site to discuss further. Participants were randomised after informed written consent had been obtained.

3b) CONSORT: Important changes to methods after trial commencement (such as eligibility criteria), with reasons

This is not applicable as there were no changes

3b-i) Bug fixes, Downtimes, Content Changes

This is not applicable as there were no bug fixes, downtime or content changes

4a) CONSORT: Eligibility criteria for participants

Eligibility criteria are included in the manuscript

4a-i) Computer / Internet literacy

This was not an appropriate eligbility criteria for this study

4a-ii) Open vs. closed, web-based vs. face-to-face assessments:

The study sites were Aberdeen Royal Infirmary and Addenbrooke’s Hospital, Cambridge. Adults (≥18 years) treated within the preceding 60 months for a previous stage 0-IIC primary cutaneous melanoma were sent information about the study, a consent form and baseline questionnaire by post.

4a-iii) Information giving during recruitment

The study sites were Aberdeen Royal Infirmary and Addenbrooke’s Hospital, Cambridge. Adults (≥18 years) treated within the preceding 60 months for a previous stage 0-IIC primary cutaneous melanoma were sent information about the study, a consent form and baseline questionnaire by post.

4b) CONSORT: Settings and locations where the data were collected

Baseline data were collected from secondary care records by a research nurse at each site before randomisation. The co-primary outcomes were Melanoma Worry Scale (MWS), anxiety and depression (HADS) and quality of life (EQ-5D-5L) [19]. Secondary outcomes were adherence to TSSE recommendations, self-efficacy and future intention and planning to perform TSSE[22]. Primary and secondary outcomes were collected by postal questionnaires at baseline, 3, 6- and 12-months after randomisation. Tertiary outcomes were new primary and recurrent melanomas and patterns of skin-related NHS resource use. These were collected 12 months after randomisation from secondary care records by research nurses blind to allocation.

4b-i) Report if outcomes were (self-)assessed through online questionnaires

Baseline data were collected from secondary care records by a research nurse at each site before randomisation. The co-primary outcomes were Melanoma Worry Scale (MWS), anxiety and depression (HADS) and quality of life (EQ-5D-5L) [19]. Secondary outcomes were adherence to TSSE recommendations, self-efficacy and future intention and planning to perform TSSE[22]. Primary and secondary outcomes were collected by postal questionnaires at baseline, 3, 6- and 12-months after randomisation. Tertiary outcomes were new primary and recurrent melanomas and patterns of skin-related NHS resource use. These were collected 12 months after randomisation from secondary care records by research nurses blind to allocation.

4b-ii) Report how institutional affiliations are displayed

This project received full approval from the North of Scotland Research Ethics Committee on 28th April 2017 ((17/NS/0040). Written informed consent was obtained from all study participants. The trial was conducted according to the principles of good clinical practice provided by Research Governance Guidelines. Consent for publication did not apply

5) CONSORT: Describe the interventions for each group with sufficient details to allow replication, including how and when they were actually administered

5-i) Mention names, credential, affiliations of the developers, sponsors, and owners

The necessary information is provided in the manuscript

5-ii) Describe the history/development process

This has been described in an earlier publication

5-iii) Revisions and updating

These items are beyond the scope if the trial report

5-iv) Quality assurance methods

Not applicable to the current study		
**5-v) Ensure replicability by publishing the source code, and/or providing screenshots/screen-capture video, and/or providing flowcharts of the algorithms used**		
These items are not relevant to the current trial report and available elsewhere		
**5-vi) Digital preservation**		
The intervention has been archieved		
**5-vii) Access**		
Briefly, intervention group participants attended a 30-minute training session at which they were issued with a seven-inch Samsung Galaxy tablet and given instruction on the intervention and how the tablet computer-base application (app) should be used to support them to conduct a thorough full-body TSSE in response to a monthly SMS text reminder sent from the trial team. The nurse demonstrated the function of the app and answered any questions about TSSE or the intervention. The app included information about the importance of monthly TSSE, instructional videos demonstrating how to perform a TSSE and take good photographs of skin lesions, a digital map of the patient's own skin, a structured checkbox list of body parts to check, prompts for the patient to plan their next TSSE and the capability to take photographs of suspicious skin lesions and send them to a dermatology nurse practitioner for review along with a text-based report of the TSSE outcomes including a description of any concerns. All participants who submitted text-based reports of any skin concerns were followed-up by the dermatology nurse practitioner.The monthly prompt was sent on a single occasion and no reminders were sent to individuals who did not complete the TSSE that month, but they would continue to be reminded on each subsequent month. The control group also completed the baseline questionnaire. All participants (intervention and control) continued to attend their usual structured melanoma follow-up as determined by local guidelines		
**5-viii) Mode of delivery, features/functionalities/components of the intervention and comparator, and the theoretical framework**		
Briefly, intervention group participants attended a 30-minute training session at which they were issued with a seven-inch Samsung Galaxy tablet and given instruction on the intervention and how the tablet computer-base application (app) should be used to support them to conduct a thorough full-body TSSE in response to a monthly SMS text reminder sent from the trial team. The nurse demonstrated the function of the app and answered any questions about TSSE or the intervention. The app included information about the importance of monthly TSSE, instructional videos demonstrating how to perform a TSSE and take good photographs of skin lesions, a digital map of the patient's own skin, a structured checkbox list of body parts to check, prompts for the patient to plan their next TSSE and the capability to take photographs of suspicious skin lesions and send them to a dermatology nurse practitioner for review along with a text-based report of the TSSE outcomes including a description of any concerns. All participants who submitted text-based reports of any skin concerns were followed-up by the dermatology nurse practitioner.The monthly prompt was sent on a single occasion and no reminders were sent to individuals who did not complete the TSSE that month, but they would continue to be reminded on each subsequent month. The control group also completed the baseline questionnaire. All participants (intervention and control) continued to attend their usual structured melanoma follow-up as determined by local guidelines		
**5-ix) Describe use parameters**		
Briefly, intervention group participants attended a 30-minute training session at which they were issued with a seven-inch Samsung Galaxy tablet and given instruction on the intervention and how the tablet computer-base application (app) should be used to support them to conduct a thorough full-body TSSE in response to a monthly SMS text reminder sent from the trial team. The nurse demonstrated the function of the app and answered any questions about TSSE or the intervention. The app included information about the importance of monthly TSSE, instructional videos demonstrating how to perform a TSSE and take good photographs of skin lesions, a digital map of the patient's own skin, a structured checkbox list of body parts to check, prompts for the patient to plan their next TSSE and the capability to take photographs of suspicious skin lesions and send them to a dermatology nurse practitioner for review along with a text-based report of the TSSE outcomes including a description of any concerns. All participants who submitted text-based reports of any skin concerns were followed-up by the dermatology nurse practitioner.The monthly prompt was sent on a single occasion and no reminders were sent to individuals who did not complete the TSSE that month, but they would continue to be reminded on each subsequent month. The control group also completed the baseline questionnaire. All participants (intervention and control) continued to attend their usual structured melanoma follow-up as determined by local guidelines		
**5-x) Clarify the level of human involvement**		
Briefly, intervention group participants attended a 30-minute training session at which they were issued with a seven-inch Samsung Galaxy tablet and given instruction on the intervention and how the tablet computer-base application (app) should be used to support them to conduct a thorough full-body TSSE in response to a monthly SMS text reminder sent from the trial team. The nurse demonstrated the function of the app and answered any questions about TSSE or the intervention. The app included information about the importance of monthly TSSE, instructional videos demonstrating how to perform a TSSE and take good photographs of skin lesions, a digital map of the patient's own skin, a structured checkbox list of body parts to check, prompts for the patient to plan their next TSSE and the capability to take photographs of suspicious skin lesions and send them to a dermatology nurse practitioner for review along with a text-based report of the TSSE outcomes including a description of any concerns. All participants who submitted text-based reports of any skin concerns were followed-up by the dermatology nurse practitioner.The monthly prompt was sent on a single occasion and no reminders were sent to individuals who did not complete the TSSE that month, but they would continue to be reminded on each subsequent month. The control group also completed the baseline questionnaire. All participants (intervention and control) continued to attend their usual structured melanoma follow-up as determined by local guidelines		
**5-xi) Report any prompts/reminders used**		
Briefly, intervention group participants attended a 30-minute training session at which they were issued with a seven-inch Samsung Galaxy tablet and given instruction on the intervention and how the tablet computer-base application (app) should be used to support them to conduct a thorough full-body TSSE in response to a monthly SMS text reminder sent from the trial team. The nurse demonstrated the function of the app and answered any questions about TSSE or the intervention. The app included information about the importance of monthly TSSE, instructional videos demonstrating how to perform a TSSE and take good photographs of skin lesions, a digital map of the patient's own skin, a structured checkbox list of body parts to check, prompts for the patient to plan their next TSSE and the capability to take photographs of suspicious skin lesions and send them to a dermatology nurse practitioner for review along with a text-based report of the TSSE outcomes including a description of any concerns. All participants who submitted text-based reports of any skin concerns were followed-up by the dermatology nurse practitioner.The monthly prompt was sent on a single occasion and no reminders were sent to individuals who did not complete the TSSE that month, but they would continue to be reminded on each subsequent month. The control group also completed the baseline questionnaire. All participants (intervention and control) continued to attend their usual structured melanoma follow-up as determined by local guidelines		
**5-xii) Describe any co-interventions (incl. training/support)**		
Briefly, intervention group participants attended a 30-minute training session at which they were issued with a seven-inch Samsung Galaxy tablet and given instruction on the intervention and how the tablet computer-base application (app) should be used to support them to conduct a thorough full-body TSSE in response to a monthly SMS text reminder sent from the trial team. The nurse demonstrated the function of the app and answered any questions about TSSE or the intervention. The app included information about the importance of monthly TSSE, instructional videos demonstrating how to perform a TSSE and take good photographs of skin lesions, a digital map of the patient's own skin, a structured checkbox list of body parts to check, prompts for the patient to plan their next TSSE and the capability to take photographs of suspicious skin lesions and send them to a dermatology nurse practitioner for review along with a text-based report of the TSSE outcomes including a description of any concerns. All participants who submitted text-based reports of any skin concerns were followed-up by the dermatology nurse practitioner.The monthly prompt was sent on a single occasion and no reminders were sent to individuals who did not complete the TSSE that month, but they would continue to be reminded on each subsequent month. The control group also completed the baseline questionnaire. All participants (intervention and control) continued to attend their usual structured melanoma follow-up as determined by local guidelines		
**6a) CONSORT: Completely defined pre-specified primary and secondary outcome measures, including how and when they were assessed**		
Baseline data were collected from secondary care records by a research nurse at each site before randomisation. The co-primary outcomes were Melanoma Worry Scale (MWS), anxiety and depression (HADS) and quality of life (EQ-5D-5L) [19]. Secondary outcomes were adherence to TSSE recommendations, self-efficacy and future intention and planning to perform TSSE[22]. Primary and secondary outcomes were collected by postal questionnaires at baseline, 3, 6- and 12-months after randomisation. Tertiary outcomes were new primary and recurrent melanomas and patterns of skin-related NHS resource use. These were collected 12 months after randomisation from secondary care records by research nurses blind to allocation.		
**6a-i) Online questionnaires: describe if they were validated for online use and apply CHERRIES items to describe how the questionnaires were designed/deployed**		
Baseline data were collected from secondary care records by a research nurse at each site before randomisation. The co-primary outcomes were Melanoma Worry Scale (MWS), anxiety and depression (HADS) and quality of life (EQ-5D-5L) [19]. Secondary outcomes were adherence to TSSE recommendations, self-efficacy and future intention and planning to perform TSSE[22]. Primary and secondary outcomes were collected by postal questionnaires at baseline, 3, 6- and 12-months after randomisation. Tertiary outcomes were new primary and recurrent melanomas and patterns of skin-related NHS resource use. These were collected 12 months after randomisation from secondary care records by research nurses blind to allocation.		
**6a-ii) Describe whether and how "use" (including intensity of use/dosage) was defined/measured/monitored**		
These are beyond the scope of the current paper and reported in a separate publication		
**6a-iii) Describe whether, how, and when qualitative feedback from participants was obtained**		
A separate qualitative manuscript has been submitted		
**6b) CONSORT: Any changes to trial outcomes after the trial commenced, with reasons**		
Baseline data were collected from secondary care records by a research nurse at each site before randomisation. The co-primary outcomes were Melanoma Worry Scale (MWS), anxiety and depression (HADS) and quality of life (EQ-5D-5L) [19]. Secondary outcomes were adherence to TSSE recommendations, self-efficacy and future intention and planning to perform TSSE[22]. Primary and secondary outcomes were collected by postal questionnaires at baseline, 3, 6- and 12-months after randomisation. Tertiary outcomes were new primary and recurrent melanomas and patterns of skin-related NHS resource use. These were collected 12 months after randomisation from secondary care records by research nurses blind to allocation.		
**7a) CONSORT: How sample size was determined**		
**7a-i) Describe whether and how expected attrition was taken into account when calculating the sample size**		
**7b) CONSORT: When applicable, explanation of any interim analyses and stopping guidelines**		

Baseline data were collected from secondary care records by a research nurse at each site before randomisation. The co-primary outcomes were Melanoma Worry Scale (MWS), anxiety and depression (HADS) and quality of life (EQ-5D-5L) [19]. Secondary outcomes were adherence to TSSE recommendations, self-efficacy and future intention and planning to perform TSSE[22]. Primary and secondary outcomes were collected by postal questionnaires at baseline, 3, 6- and 12-months after randomisation. Tertiary outcomes were new primary and recurrent melanomas and patterns of skin-related NHS resource use. These were collected 12 months after randomisation from secondary care records by research nurses blind to allocation.		
**8a) CONSORT: Method used to generate the random allocation sequence**		
Participants were randomised 1:1 to intervention or control using a remote automated computer-allocated application hosted at the Centre for Healthcare Randomised Trials (CHaRT) in Aberdeen, UK. An algorithm minimised imbalance in sex and centre between groups[21]. Due to the nature of the intervention, both participants and researchers were not masked to randomised allocation.		
**8b) CONSORT: Type of randomisation; details of any restriction (such as blocking and block size)**		
Participants were randomised 1:1 to intervention or control using a remote automated computer-allocated application hosted at the Centre for Healthcare Randomised Trials (CHaRT) in Aberdeen, UK. An algorithm minimised imbalance in sex and centre between groups[21]. Due to the nature of the intervention, both participants and researchers were not masked to randomised allocation.		
**9) CONSORT: Mechanism used to implement the random allocation sequence (such as sequentially numbered containers), describing any steps taken to conceal the sequence until interventions were assigned**		
Participants were randomised 1:1 to intervention or control using a remote automated computer-allocated application hosted at the Centre for Healthcare Randomised Trials (CHaRT) in Aberdeen, UK. An algorithm minimised imbalance in sex and centre between groups[21]. Due to the nature of the intervention, both participants and researchers were not masked to randomised allocation.		
**10) CONSORT: Who generated the random allocation sequence, who enrolled participants, and who assigned participants to interventions**		
Participants were randomised 1:1 to intervention or control using a remote automated computer-allocated application hosted at the Centre for Healthcare Randomised Trials (CHaRT) in Aberdeen, UK. An algorithm minimised imbalance in sex and centre between groups[21]. Due to the nature of the intervention, both participants and researchers were not masked to randomised allocation.		
**11a) CONSORT: Blinding - If done, who was blinded after assignment to interventions (for example, participants, care providers, those assessing outcomes) and how**		
**11a-i) Specify who was blinded, and who wasn't**		
Blinding was not possible in this study		
**11a-ii) Discuss e.g., whether participants knew which intervention was the "intervention of interest" and which one was the "comparator"**		
participant knew if they were in the intervention or control group		
**11b) CONSORT: If relevant, description of the similarity of interventions**		
There was only one intervention trialled		
**12a) CONSORT: Statistical methods used to compare groups for primary and secondary outcomes**		
A statistical analysis section is provided in the manuscript		
**12a-i) Imputation techniques to deal with attrition / missing values**		
These were not employed in the current study		
**12b) CONSORT: Methods for additional analyses, such as subgroup analyses and adjusted analyses**		
There were no subgroup analyses conducted		
RESULTS		
**13a) CONSORT: For each group, the numbers of participants who were randomly assigned, received intended treatment, and were analysed for the primary outcome**		
This information is within the results section and tables		
**13b) CONSORT: For each group, losses and exclusions after randomisation, together with reasons**		
A study flow diagram is included in the manuscript		
**13b-i) Attrition diagram**		
A study flow diagram is included in the manuscript		
**14a) CONSORT: Dates defining the periods of recruitment and follow-up**		
Between 24 January 2018 and 8 March 2019, 240 participants were randomised (121 to the ASICA intervention,119 to usual care).		
**14a-i) Indicate if critical "secular events" fell into the study period**		
This is not relevant to the current study		
**14b) CONSORT: Why the trial ended or was stopped (early)**		
This is not relevant to the current study		
**15) CONSORT: A table showing baseline demographic and clinical characteristics for each group**		
This is included in the manuscript		
**15-i) Report demographics associated with digital divide issues**		
Demographics are reported in the paper		
**16a) CONSORT: For each group, number of participants (denominator) included in each analysis and whether the analysis was by original assigned groups**		
**16-i) Report multiple "denominators" and provide definitions**		
This information is reported in the paper		
**16-ii) Primary analysis should be intent-to-treat**		
The analysis was by the intention-to-treat principle		
**17a) CONSORT: For each primary and secondary outcome, results for each group, and the estimated effect size and its precision (such as 95% confidence interval)**		
This information is reported in the results section and the tables		
**17a-i) Presentation of process outcomes such as metrics of use and intensity of use**		
This information has been presented in another publication		
**17b) CONSORT: For binary outcomes, presentation of both absolute and relative effect sizes is recommended**		
This information is reported in the paper		
**18) CONSORT: Results of any other analyses performed, including subgroup analyses and adjusted analyses, distinguishing pre-specified from exploratory**		
All relevant analyses are reported in the paper		
**18-i) Subgroup analysis of comparing only users**		
This is not relevant to the current study		
**19) CONSORT: All important harms or unintended effects in each group**		
This information is reported in the manuscript		
**19-i) Include privacy breaches, technical problems**		
This is not relevant to the current manuscript		
**19-ii) Include qualitative feedback from participants or observations from staff/researchers**		
Qualitative feedback is being reported in another related publication		
DISCUSSION		
**20) CONSORT: Trial limitations, addressing sources of potential bias, imprecision, multiplicity of analyses**		
**20-i) Typical limitations in ehealth trials**		
We provide a structured discussion in the paper		
**21) CONSORT: Generalisability (external validity, applicability) of the trial findings**		
**21-i) Generalizability to other populations**		
We provide a structured discussion in the paper		
**21-ii) Discuss if there were elements in the RCT that would be different in a routine application setting**		
We provide a structured discussion in the paper		
**22) CONSORT: Interpretation consistent with results, balancing benefits and harms, and considering other relevant evidence**		
**22-i) Restate study questions and summarize the answers suggested by the data, starting with primary outcomes and process outcomes (use)**		
We provide a structured discussion in the paper		
**22-ii) Highlight unanswered new questions, suggest future research**		
We provide a structured discussion in the paper		
Other information		
**23) CONSORT: Registration number and name of trial registry**		
This information is provided		
**24) CONSORT: Where the full trial protocol can be accessed, if available**		
The full protocol is reference in the paper		
**25) CONSORT: Sources of funding and other support (such as supply of drugs), role of funders**		

This information is provided in the manuscript		
**X26-i) Comment on ethics committee approval**		
This project received full approval from the North of Scotland Research Ethics Committee on 28th April 2017 ((17/NS/0040). Written informed consent was obtained from all study participants. The trial was conducted according to the principles of good clinical practice provided by Research Governance Guidelines. Consent for publication did not apply		
**x26-ii) Outline informed consent procedures**		
Those interested in participating in the study were contacted by the recruiting site to discuss further. Participants were randomised after informed written consent had been obtained.		
**X26-iii) Safety and security procedures**		
These are not specifically discussed in the current trial report		
**X27-i) State the relation of the study team towards the system being evaluated**		
This information is provided in the manuscript		
